# Development and Characterisation of Composites Prepared from PHBV Compounded with Organic Waste Reinforcements, and Their Soil Biodegradation

**DOI:** 10.3390/ma17030768

**Published:** 2024-02-05

**Authors:** Valentin Furgier, Andrew Root, Ivo Heinmaa, Akram Zamani, Dan Åkesson

**Affiliations:** 1 Swedish Centre for Resource Recovery, University of Borås, 501 90 Borås, Sweden; vvalentin.furgier@etu.uca.fr (V.F.); akram.zamani@hb.se (A.Z.); 2 MagSol, Tuhkanummenkuja 2, 00970 Helsinki, Finland; magsol@kolumbus.fi; 3 National Institute of Chemical Physics and Biophysics, 12618 Tallinn, Estonia; ivo.heinmaa@gmail.com

**Keywords:** PHBV, biocomposite, biodegradation, sawdust, hair waste, chitin

## Abstract

Poly(3-hydroxybutyrate-co-3-hydroxyvalerate) (PHBV) is a biobased and biodegradable polymer. This polymer is considered promising, but it is also rather expensive. The objective of this study was to compound PHBV with three different organic fillers considered waste: human hair waste (HHW), sawdust (SD) and chitin from shrimp shells. Thus, the cost of the biopolymer is reduced, and, at the same time, waste materials are valorised into something useful. The composites prepared were characterised by differential scanning calorimetry (DSC), thermogravimetric analysis (TGA), tensile strength and scanning electron micrograph (SEM). Tests showed that chitin and HHW did not have a reinforcing effect on tensile strength while the SD increased the tensile strength at break to a certain degree. The biodegradation of the different composites was evaluated by a soil burial test for five months. The gravimetric test showed that neat PHBV was moderately degraded (about 5% weight loss) while reinforcing the polymer with organic waste clearly improved the biodegradation. The strongest biodegradation was achieved when the biopolymer was compounded with HHW (35% weight loss). The strong biodegradation of HHW was further demonstrated by characterisation by Fourier-transform infrared spectroscopy (FTIR) and solid-state nuclear magnetic resonance (NMR). Characterisation by SEM showed that the surfaces of the biodegraded samples were eroded.

## 1. Introduction

Fossil-based plastics contribute to environmental pollution, and they are generally not biodegradable. It has been estimated that 6.3 billion tons of plastics were produced between 1950 and 2015, with only 9% of this volume being recycled [1]. Consequently, there is a focus on the development of biodegradable polymers. Promising biopolymers include polylactic acid (PLA), polybutylene succinate (PBS) and thermoplastic starch (TPS), and blends and biocomposites from these plastics are under development [2,3,4].

Polyhydroxyalkanoates (PHAs) are a family of polyesters produced by microorganisms (such as Bacillus megaterium) as a way of storing energy. The most common polymer in the PHA family is polyhydroxyalkanoate (PHB), which can be produced from biomass and is well known to be biodegradable in many different climates [5,6]. PHB is, however, a rather brittle polymer and, for some applications, less brittle materials are needed. The copolymer polyhydroxybutyrate-co-valerate (PHBV) is less brittle than PHB and can therefore be a better option. However, both polymers are rather expensive, which has hampered their usage in packaging applications, so PHAs are presently only being used on the market in relatively small volumes.

Commercial plastics are often compounded with inorganic fillers such as chalk and talc which may considerably lower the cost but also improve some technical properties such as stiffness [7,8]. However, organic waste materials may be also used instead of inorganic fillers. Thus, waste materials with little or no commercial value can be valorised into useful materials while significantly reducing the cost of the biopolymer. This could potentially be important for high-volume products such as various packaging applications.

Many different types of organic waste are being generated in society which need to be valorised into materials or fuels. Thus, compounding thermoplastics with organic waste can serve the purpose of reducing the cost of the biopolymer while the filler is valorised into a new material. Wood flour and sawdust (SD) are already used to produce wood polymer composites, typically together with traditional polymers such as polypropylene and polyethylene [9,10]. Wood flour has also been evaluated as a filler for biopolymers such as PLA [11], PHB [12] and polybutylene succinate (PBS) [13]. Chitin is one of the most abundant polymers in nature. It is a polysaccharide found in the cell wall of fungi and in shells of crustaceans. Crustacean shells are generated in large volumes by the food industry, which is a waste of low commercial value, leaving the valuable chitin underutilised. Furthermore, due to the lack of adequate end-of-life disposal options, seafood waste is often incinerated, landfilled or dumped in the sea [14].

Another type of organic waste is human hair waste (HHW), which consists mainly of keratin protein and is produced in relatively large quantities. No statistics are, to our knowledge, available, but Gupta estimated the annual human hair waste in India alone to be roughly 120 million kg [15]. Presently, this type of waste has very little use and could possibly be used as a filler for polymers, but this is presently not well studied. Although few studies have examined the addition of HHW to polymeric materials, the addition of human hair to polypropylene has been studied by Choudhry and Pandey [16].

The aim of this study was to reduce the cost of BHBV by compounding it with PHBV, with HHW, chitin and SD, simultaneously valorising these waste materials into a useful material. However, the addition of waste materials may alter the properties of the neat polymer. Therefore, the thermal and mechanical properties as well as the biodegradation of the composites were investigated. The biodegradation was assessed using a soil degradation test over five months. The degraded compounds were monitored by gravimetric, thermal and spectroscopic methods.

## 2. Materials and Methods

### 2.1. Materials

The PHBV (ENMAT PHBV resin Y1000P) was delivered by TianAn Biopolymer (Ningbo, China). Softwood sawdust (particles ranging up to a few mm, from a local sawmill outside Borås, Sweden), chitin, a fine powder from shrimp shells (Sigma Aldrich, St. Louis, MO, USA, practical grade), and HHW (harvested from a local hairdressing salon, Borås, Sweden) were used as fillers. A microscope measurement shows a diameter between 70 µm and 100 µm and a length around 1 cm.

### 2.2. Extrusion-Moulding

Prior to processing, the materials were dried at 50 °C for 8 h under vacuum. PHBV was then compounded with organic waste materials reinforcement using a 15 mL twin-screw micro-compounder (DSM Xplore, Sittard, The Netherlands). Two co-rotating screws (the diameter was 20 mm, and the length was 173 mm) were used. Samples were compounded using a temperature profile of 180/180/185 °C at 70 rpm for three minutes. Test bodies for tensile tests (75 mm long) were prepared by transferring the plastic melt to a lab-scale injection moulding instrument from the same company. The mould temperature was 110 °C.

The different qualities of filler were prepared with 13 wt.% or 30.0 wt.%

### 2.3. Characterisation

DSC analysis (Q2000 from TA Instruments, New Castle, DE, USA) was performed to determine the thermal properties using dynamic scans in an atmosphere of nitrogen. The samples were heated from −40 °C to 200 °C using a heating speed of 10 °C/min. Glass transition temperature (Tg), peak of the melting temperature (Tm) and heat of fusion (ΔHM) were recorded from the second heating. The crystallinity (Xc) was determined from the second heating using the following equation:

$$Xc=\Delta Hm/{\Delta H}_{M}^{0}\times 100/\Phi$$

where Φ is the weight fraction of the compounded filler, ΔHm is the melting enthalpy and ${\Delta H}_{M}^{0}$ is the heat of fusion for the 100% crystalline polymer. A value for ${\Delta H}_{M}^{0}$ of 146 J/g was used [17].

Samples were characterised by TGA using a TGA Q500 from TA Instruments. Samples were heated at 10 °C/min until 800 °C under an atmosphere of nitrogen. The temperature at 5% weight loss (T5), the peak of the derivative weight curve (TMAX) and the residual weight were recorded. Characterisation with DCS and TGA was performed in duplicate.

Biodegraded samples were characterised by Fourier transform infrared spectroscopy (FTIR, Nicolet iS10, Thermo Fischer Scientific, Waltham, MA, USA) equipped with an attenuated total reflectance unit (ATR). Spectra were recorded from 400 to 4000 cm^−1^ and 64 scans to determine whether there was any chemical reaction on the surface of the samples.

The tensile properties were evaluated by tensile test using a H10KT Tinius Olsen. Dog bone-shaped test bodies (Tinius Olsen, Horsham, PA, USA), 75 mm long, were fractured using a test speed of 10 mm/min and a 5000 N load cell. A minimum of five test bodies were tested.

^13^C MAS NMR spectra were recorded on a AVANCE-II spectrometer (Bruker Corporation, Billerica, MA, USA) in a 14.1 T magnetic field (^13^C resonance frequency 150.9 MHz) using a home-built MAS NMR probe for 25 × 4 mm Si_3_N_4_ rotors. In all experiments, the sample spinning frequency was set to 12.5 kHz. The NMR spectra were recorded with proton decoupling (H1 = 100 kHz) using cross polarisation (CP) with a 2 ms ramped spin locking pulse in the 1H channel together with a pulse in the 13C channel of suitable amplitude. The relaxation delay in the CPMAS NMR experiments was 5 s and the number of transients acquired was 2000. The direct excitation experiment, using a single pulse on the ^13^C channel with 1H decoupling, was conducted with a 90° pulse (3 μs), a 60 s relaxation delay and 1000 transients.

Scanning electron micrography (SEM) was used to characterise the fractured surfaces after tensile tests as well as test bodies after biodegradation. Characterisation by SEM was performed by an external institute.

### 2.4. Biodegradation

The biodegradation was evaluated with soil degradation tests. Neat PHBV and PHBV compounded with 13 wt.% of the different fillers were evaluated. The test was conducted in flowerpots. Each pot was filled with 880 g of flower soil. The test bodies, about 15 × 10 × 2 mm, were buried at a depth of approximately 10 cm. The pots were covered with aluminium foil and kept indoors. The average temperature during the test was 21.5 °C and water content in soil was adjusted to 40 wt.% every third week. The biodegradation was monitored by gravimetric tests. The samples were rinsed in water and wiped with tissue paper. The samples were finally dried in a vacuum oven for 1 h at 50 °C under vacuum before being weighed. After biodegradation, the test bodies were also characterised by SEM, FTIR and solid-state NMR.

## 3. Results and Discussions

### 3.1. Thermal Characterisation

PHBV was compounded with SD, HHW and chitin and the result of the thermal characterisation by DSC is summarised in Table 1. Typical examples of the characterisation by DSC are shown in Figure S1. The neat polymer had a crystallinity of about 63% while the glass transition temperature is not clearly seen. Adding the fillers to the polymer matrix only changed the crystallinity to a small degree and there was no significant difference between the samples. As an example, adding 30% chitin to the polymer changed the crystallinity from 62.9% to 61.3%. Using the *t*-test (*p* = 0.05) there is no significant difference between the two values. Srubar et al. blended PHBV with wood flour. When adding 36 vol-% untreated wood flour, the crystallinity was reduced by less than 10% [18].

The samples were also characterised by TGA; the results are summarised in Table 2 and examples of thermograms are shown in Figure S2. The neat polymer lost 5 wt.% at 269°, displaying a maximum degradation temperature at 294 °C. The polymer was degraded at 400 °C with a residue of 1.3 wt.%. This is largely in agreement with Corre et al. [19]. The degradation occurs by chain scission and hydrolytic cleavage with oligomers and crotonic acid as a degradation product [20,21].

Adding the fillers to the polymer changed the thermal degradation to a small degree. Adding 30 wt.% SD to the polymer matrix changed the thermal stability to a very small degree. For example, adding 30% SD to the polymer matrix changed T_5_ from 268.9 °C to 269.3 °C. However, a second peak of the derivate weight appears at 371 °C. This is caused by the degradation of the wood fibres [22]. Vandi et al. compounded PHBV with wood fibres. A decreased onset was recorded with increased content of the wood fibre [23].

The addition of HHW more clearly reduced the thermal stability. T_5_ was reduced from about 269 °C to 253 °C and the maximum degradation temperature ranged between 293 °C and 287 °C. Arslan et al. characterised keratin from human hair by TGA. At 250 °C, a weight loss of more than 20 wt.% was observed. As HHW consists mainly of keratin, this could explain the lower thermal stability.

### 3.2. Tensile Strength

The results of tensile testing are shown in Table 3. Typical examples from the tensile testing can be seen in Figure S3. The neat polymer has a tensile strength at break of 34.5 MPa and a modulus of 1.7 GPa. Compounding the polymer with SD reinforced the polymer. At 30 wt.% of the filler, the tensile strength at break increased to 46.2 MPa and the modulus to 2.7 GPa. As expected, adding an SD to PHBV clearly reduced the elongation at break, from 13.9% to 2.9%. A similar pattern was observed by Vandi et al. [23]. Chitin did not significantly reinforce PHBV. Adding 13% chitin increased the tensile strength to 37 MPa but the increase is not significant. The mechanical properties could probably be improved by the use of a coupling agent or a surface modification of the filler. Wang et al. prepared surface modified chitin nanocrystals by acetylation. The crystals were mixed into PHBV by solution casting [24]. The chitin nanocrystals had a relatively strong reinforcing effect. As for the HHW, the filler clearly also had no reinforcing effect. A proportion of 30% HHW actually reduced the tensile strength somewhat. HHW mainly consists of protein with highly polar side groups while PHBV is more hydrophobic. Without surface modification or the use of a coupling agent, this filler is therefore not expected to give a reinforcing effect. Although other protein sources are commonly used as fillers for thermoplastic resins, HHW as a filler is not well studied. However, studies with hydrolysed keratin as a filler has been done. For example, Danko et al. compounded a plasticised blend of polylactic acid (PLA) and PHBV with hydrolysed keratin. A clear reduction of the mechanical properties was observed with increased keratin content [25].

**Figure 1:**
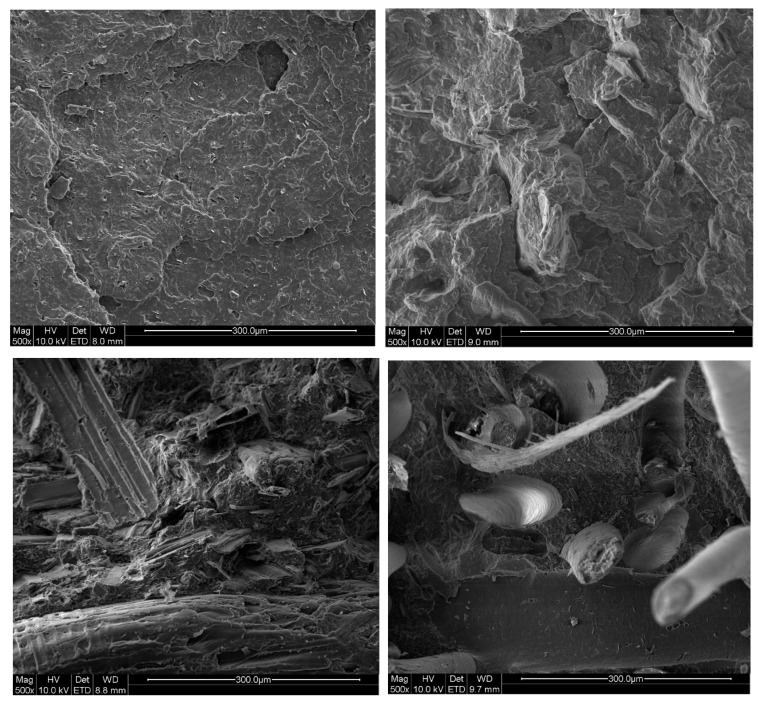
SEM micrographs of the fractured surfaces of neat PHBV (**top left**), chitin 30% (**top right**), SD 30% (**bottom left**) and HHW 30% (**bottom right**).

The morphology of the fractured test bodies was characterised by SEM. The micrographs can be seen in Figure 1. For chitin, cracks can be seen between chitin and the polymer matrix. This indicates a low adhesion between the filler and the polymer. For SD, fibre bundles can be seen being pulled out of the matrix which also indicates low adhesion. Also, for HHW, cracks between the fibres and the polymer matrix can be seen as well as pulled-out fibres. The use of a coupling agent or a surface modification of the filler could most likely have improved the adhesion [24].

### 3.3. Biodegradation

**Figure 2:**
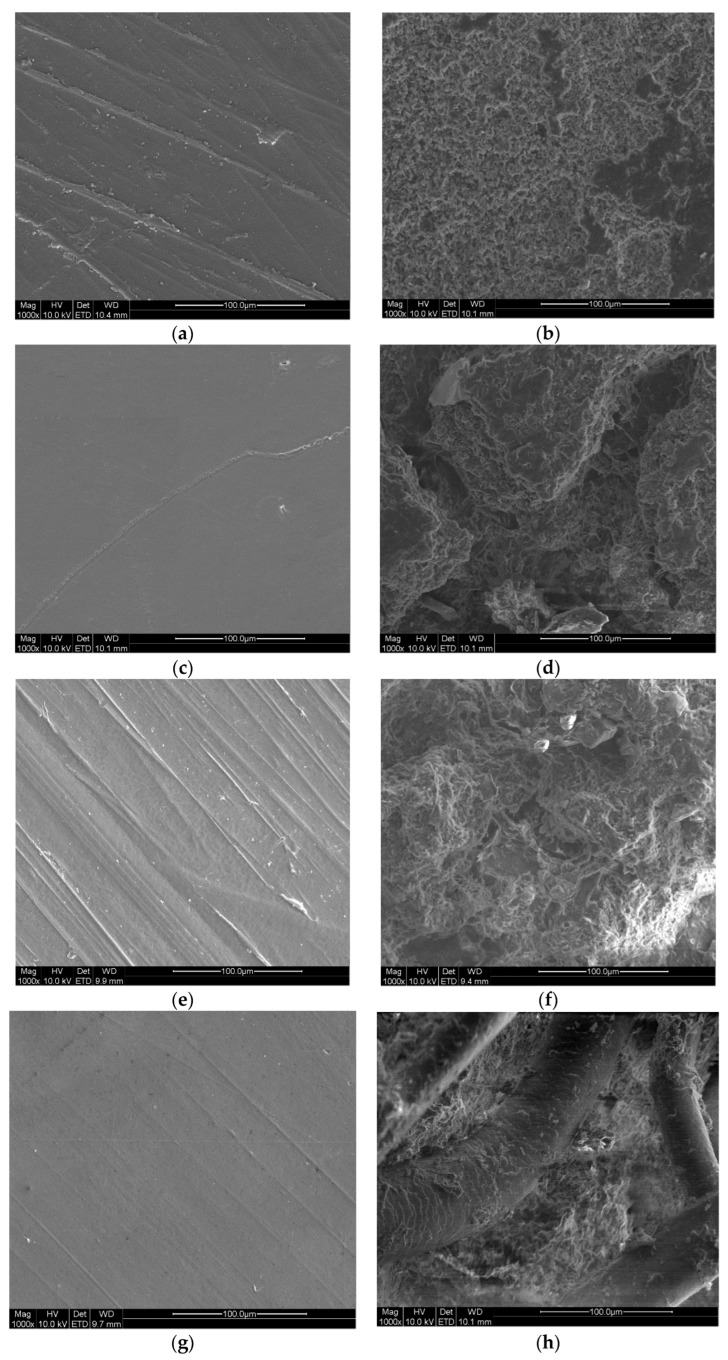
SEM micrographs of the PHBV and its composites before and after five months biodegradation: (**a**) neat PHBV before biodegradation, (**b**) neat PHBV after biodegradation, (**c**) SD before biodegradation, (**d**) SD after biodegradation, (**e**) chitin before biodegradation, (**f**) chitin after biodegradation, (**g**) HHW before biodegradation and (**h**) HHW after biodegradation.

The biodegradation of the prepared composites was evaluated with a soil degradation test over five months. The biodegradation was monitored by gravimetry, FTIR, SEM and solid-state NMR. The SEM micrographs of neat PHBV and PHBV with fillers can be observed in Figure 2. Before degradation, the surfaces of the specimens are smooth, while after biodegradation they appear rough with many cavities. Neat PHBV had a smooth surface before biodegradation but after five months the surface was eroded. In comparison to neat PHBV, SD was more degraded, with large cavities on the surface. As for chitin, a relatively strong surface erosion can be seen. HHW was also heavily eroded with clearly visible individual fibres uncovered from the polymer matrix.

**Figure 3:**
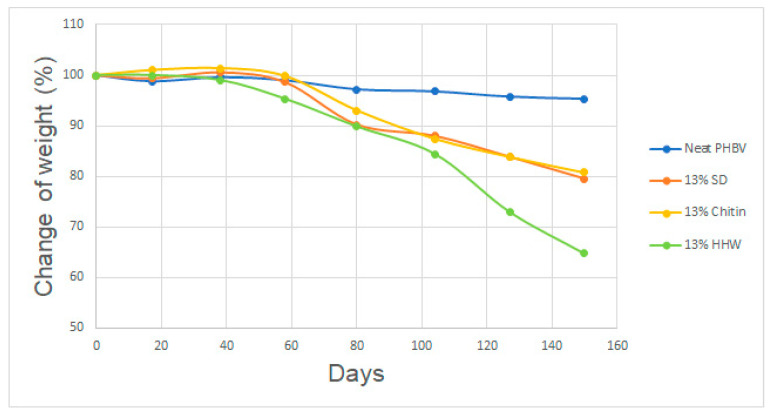
Change of weight (%) of the test bodies plotted against soil burial time.

The result of the gravimetric test is shown in Figure 3. It is interesting to note that the neat PHBV only degraded to a small degree while test bodies with fillers degraded faster. After five months, the weight loss was only about 5% for neat PHBV. Teramoto et al. studied soil degradation of PHBV. After 150 days, a weight loss of roughly 30% was recorded [26]. This is clearly higher than what was found in this study. A difference is that thin films (0.5 mm thickness) were used in that study while 2 mm thick test bodies were used in this study. It is known that PHBV is degraded by surface erosion [27,28]. In other words, water cannot easily penetrate the polymer and erosion and hydrolysis mainly take place on the surface while the bulk remains unaltered. As a consequence, thick test bodies will inevitably show a lower percentual reduction of the weight.

Adding organic fillers to the polymer matrix clearly improved the biodegradation. When SD and chitin were added to the polymer matrix, the weight loss was roughly 20% after five months. Rosdi and Zakaria prepared PLA/chitin composites by solution casting with fibre loadings up to 4 phr. Samples were buried in compost soil for 10 weeks and it was found that the biodegradation increased with increasing content of chitin [29].

The biodegradation in soil of various polymers such as PHB [30], PLA [31] and PLA/starch [32] compounded with wood flour have been evaluated under various conditions. A general trend is that the biopolymers biodegrade faster with an increased level of the wood-flour content. Wood is hygroscopic, so adding wood flour to the more hydrophobic matrix will increase the biodegradation.

Samples compounded with HHW displayed the highest weight loss. After five months of biodegradation, the samples had lost about 35% of their initial weight. Since neat PHBV only degraded by 5 wt.% and the content of HHW was only 13 wt.%, it is clear that the inclusion of HHW notably increased the degradation and that the inclusion of HHW also increased the degradation of PHBV. It is possible that the nitrogen in keratin may improve the biodegradation [33]. Moreover, proteins contain many hydrophilic groups such as carboxylic groups and are therefore hygroscopic. Thus, the inclusion of HHW may also increase the water absorption and the biodegradation rate increases as a consequence.

**Figure 4:**
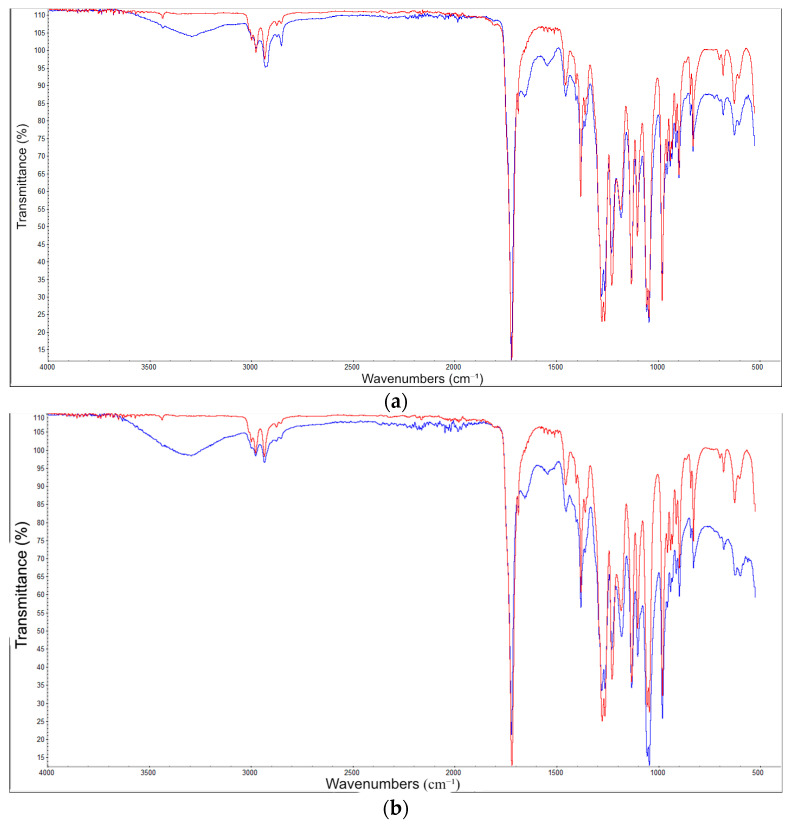

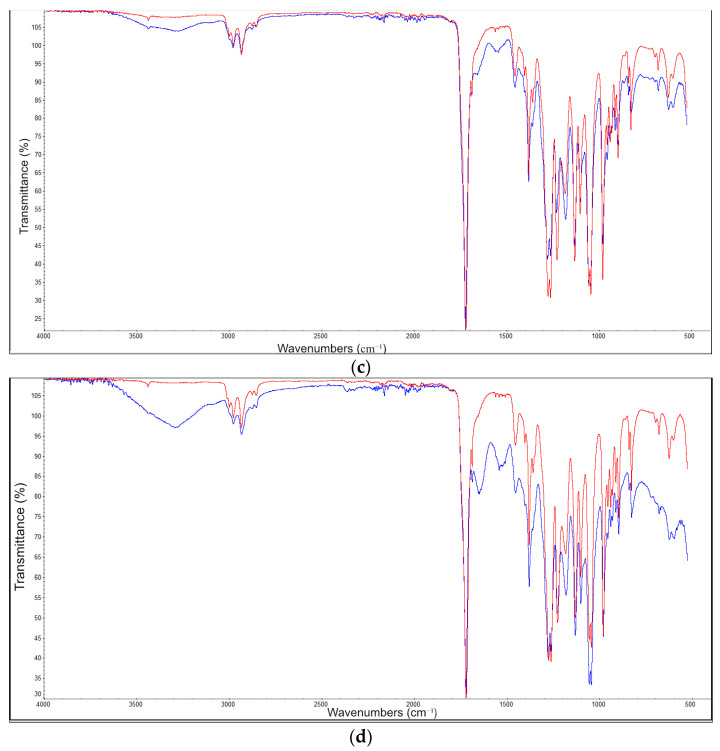
FTIR spectra for (**a**) neat PHBV, (**b**) 13 wt.% SD, (**c**) 13 wt.% chitin and (**d**) 13 wt.% HHW before (red) and after five months of biodegradation (blue).

The biodegraded samples were also characterised by FTIR, Figure 4. The spectrum for neat PHBV (a) only changed to a small degree after biodegradation. Chitin and SD showed signs of some biodegradation with changes at 3300 cm^−1^. HHW, in stark contrast to neat PHB, displayed signs of strong degradation. A strong band is seen at about 3300 cm^−1^ and the band at 1700 cm^−1^ has almost disappeared, demonstrating that the polyester has been heavily degraded. The band at about 3300 cm^−1^ is assigned to stretching of the hydroxyl groups (O-H) and it can be seen that this band has increased. This is caused by hydrolytic cleavage of the polyester. A new peak emerges at 1650 cm^−1^. Carbon-carbon stretch is often observed at about 1645 cm^−1^. However, characterisation by NMR did not support any unsaturated carbon-carbon bonds (see the NMR characterisation below). Therefore, the band at 1650 cm^−1^ is assigned the C=O stretch (Amide I) [34]. There is also a new, large absorption seen at 1550 cm-1 which also comes from proteins (Amide II) [34]. The same two bands can also be observed in the spectra of the other biodegraded test bodies. N-H bonds are not expected in the structure of, for example, neat PHBV. Therefore, the appearance of the two bands at 1650 cm^−1^ and 1550 cm^−1^ must be ascribed to the formation of a microbial film on the surface of the biodegraded test bodies.

**Figure 5:**
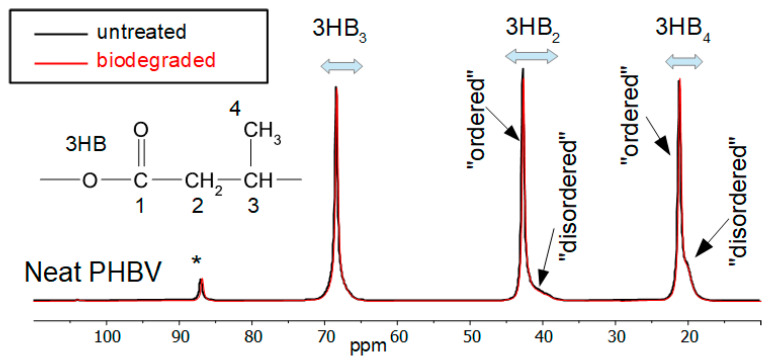
^13^C CPMAS NMR spectra of neat PHBV before and after biodegradation. * indicates the peak positions of the MAS spinning sidebands.

Test bodies before and after five months of biodegradation were also characterised by NMR. Figure 5 shows the ^13^C CPMAS spectra of the neat polymer before and after biodegradation.

In both spectra, the ordered (associated with the crystalline region) and disordered (associated with the amorphous regions) parts of the polymer matrix can be seen as narrow and broad peaks, respectively, for each carbon observed [35]. The peaks labelled as ordered and disordered are related to the crystalline and amorphous regions of the polymer but may not agree exactly with other techniques, such as DSC, since NMR measures order on a smaller scale.

**Figure 6:**
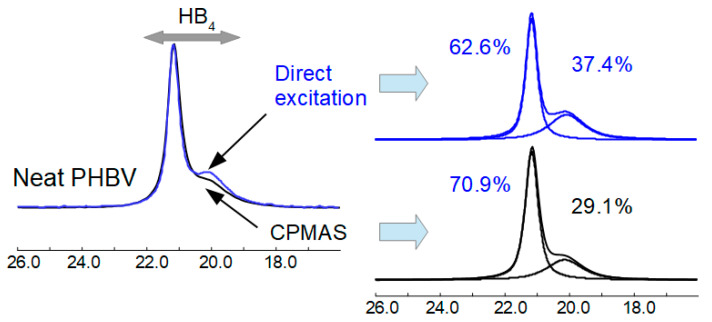
Comparison of the direct excitation and CPMAS NMR spectra of the methyl area of the PHBV polymer.

Biodegradation does not appear to significantly change the morphology of the pure polymer according to the solid-state NMR spectrum. Due to time restrictions, it was not possible to run all samples using the direct excitation experiment (which is quantitative for the PHBV methyl peak [36]). To estimate the ordered/disordered amounts of PHBV polymer quantitatively, a comparison was made of the ordered/disordered methyl peak amounts around 20 ppm from the direct excitation and CPMAS NMR spectra of three of the samples. From this comparison, it was possible to calculate a scaling factor for the amounts obtained from the CPMAS NMR spectra which should reflect the true number of these species. An example is shown in Figure 6 of the comparison of the direct excitation and CPMAS NMR spectra for the pure PHBV polymer.

Using the scaling factor obtained, the relative amount of ordered and disordered PHBV polymer was calculated using the methyl peak of PHBV from the CPMAS NMR spectra. These results are shown in Table 4.

**Figure 7:**
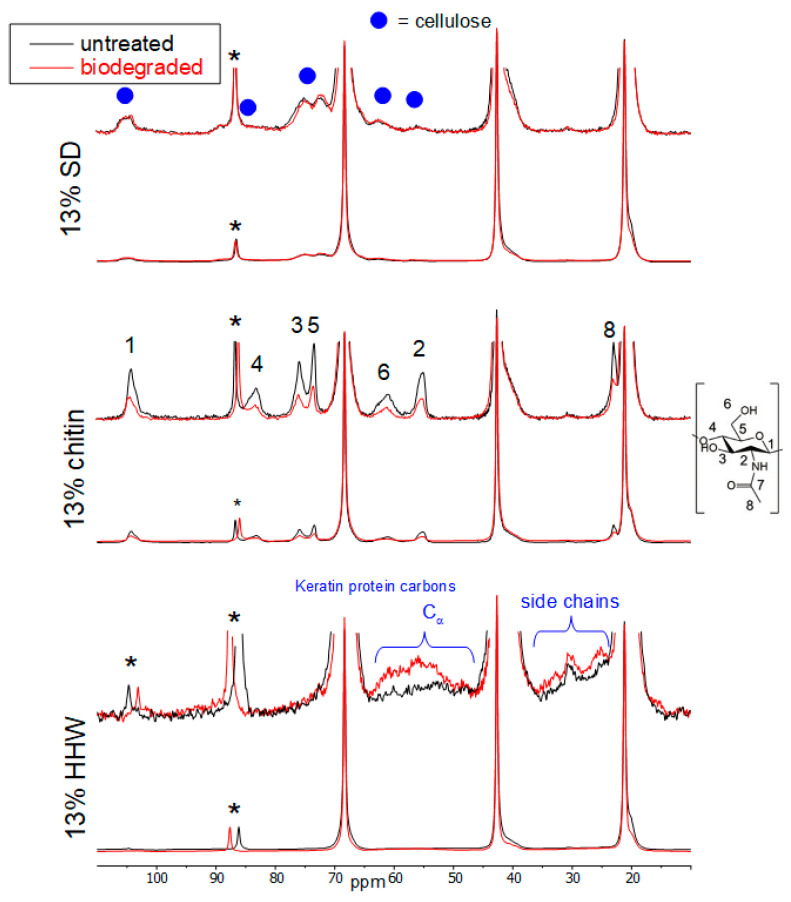
^13^C CPMAS NMR spectra of the 13% SD, chitin and HHW samples before and after biodegradation. Spectra are normalised to the height of the ordered PHBV methyl peak at 21 ppm. * indicates the peak positions of the MAS spinning sidebands. The blue circles indicate the peaks due to cellulose.

Figure 7 shows the ^13^C CPMAS NMR spectra of the 13% SD, chitin and HHW samples. The spectra were normalised to the ordered peak of the methyl from the PHBV. The SD sample does not show any large differences after biodegradation except for a change in the relative amounts of ordered and disordered PHBV, as shown in Table 4. A less-disordered polymer appears to be present after biodegradation. Since only 13 wt.% of SD is present and we observe a 20 wt.% loss, but the relative amounts of SD (cellulose part) and PHBV are similar after biodegradation; it seems likely that there may have been degradation of both the polymer and the SD.

The reduced amount of disordered PHBV after biodegradation may also indicate that degradation of the PHBV preferentially occurs within the amorphous region. The ^13^C NMR peaks due to any lignin present in the SD sample were too weak to draw any significant conclusions and it is not possible to say if degradation of that part of the SD also occurred.

The 13 wt.% chitin sample shows a reduced amount of chitin, relative to the PHBV polymer, after degradation. In this case, the percentages of ordered and disordered PHBV regions are similar before and after biodegradation (Table 4). This sample also showed a 20 wt.% loss even though only 13 wt.% of chitin was present, and some of this was still present after biodegradation. Therefore, it is concluded that degradation occurs in both components. However, for this chitin sample, there is more degradation of the filler than for the SD sample above. Also, the fact that the ordered/disordered amounts of PHBV are similar before and after biodegradation indicates that degradation occurs in all regions of the PHBV polymer, irrespective of the morphology.

The 13 wt.% HHW sample shows the biggest weight loss (35%) but an increase in the relative intensities of the HHW peaks in the solid-state NMR spectrum compared to the PHBV polymer after biodegradation. Since the amount of HHW cannot increase after degradation, this means that there has been a significant loss of the PHBV polymer after biodegradation. This sample also shows the biggest change in PHBV polymer morphology, with a much lower amount of disordered PHBV polymer present after biodegradation (Table 4), indicating that much of this loss probably occurs within the disordered domains of the PHBV polymer. The shape of the Cα carbon peaks from the keratin proteins changed somewhat after biodegradation which may also indicate that they play a part in the degradation process. It is impossible to determine the exact nature of the change in the different protein groups due to the poor signal-to-noise ratio in this area and the complexity of the proteins present.

**Figure 8:**
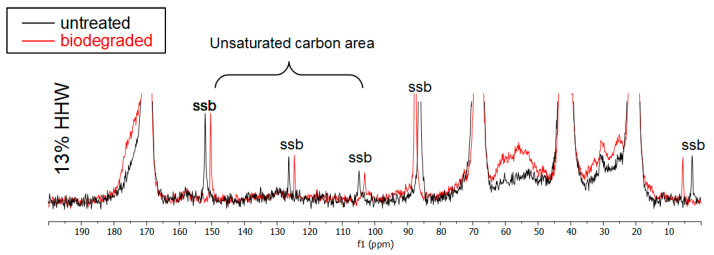
Vertical expansion of the ^13^C CPMAS NMR spectra of the 13% HHW sample before and after biodegradation showing the whole spectrum. The MAS spinning speeds were slightly different, hence their different positions in each spectrum.

FTIR showed that the biodegraded sample, and especially HHW, possibly had absorption bands from some unsaturated carbons but NMR was unable to confirm this since this area had weak spinning sidebands (ssb) and other small broad peaks in both the untreated and biodegraded HHW sample (Figure 8).

## 4. Conclusions

PHBV was compounded with organic waste materials. Adding chitin and HHW to the polymer matrix did not improve the mechanical properties, while the addition of SD increased the tensile strength at break from 36 MPa to 46 MPa. The addition of fillers to the PHBV did not significantly change the crystallinity.

The biodegradation of the prepared composites was evaluated with a soil burial test. Test bodies were buried in flower soil for five months and the biodegradation was monitored gravimetrically. This revealed that the neat PHBV degraded rather slowly but when the fillers were added, the biodegradation was clearly improved. The highest biodegradation was recorded when the polymer was compounded with HHW. Characterisation by FTIR and NMR also confirmed that the samples were degraded.

HHW clearly gave the fastest biodegradation. HHW could possibly be added to PHBV for some applications as a cheap filler that improves biodegradation. More studies would be needed to investigate possible effects of hair types, hair treatments and possible ways of increasing the adhesion between HHW and the polymer matrix.

PHBV is today a rather expensive polymer sold in small quantities. Compounding the polymer with cheap fillers can not only lower the cost of PHBV, but, at the same time, waste materials are valorised into a useful material. An additional benefit of the addition of waste is that the biodegradation of PHBV in soil can be improved.

## Figures and Tables

**Table 1:** Thermal properties after characterisation by DSC. The standard deviation is given in parentheses.

Sample	ΔHm (J/g)	Xc (%)
Neat PHBV	91.9 (2.1)	62.9 (1.4)
SD 13%	76.1 (1.8)	61.3 (1.8)
SD 30%	66.5 (0.5)	65.1 (0.5)
HHW 13%	81.7 (1.0)	65.9 (1.0)
HHW 30%	67.0 (0.2)	65.5 (0.2)
Chitin 13%	79.0 (1.3)	63.7 (1.3)
Chitin 30%	62.7 (1.2)	61.3 (0.4)

**Table 2:** Characterisation by TGA. The standard deviation is given in parentheses.

Sample	T_5_ (°C)	T_MAX1_ (°C)	T_MAX2_ (°C)	Residue wt.%
Neat PHBV	268.9 (3.2)	293.9 (0.3)	-	1.3 (0.1)
SD 30%	269.3 (9.8)	296.3 (5.8)	371.0 (1.8)	6.1 (0.0)
HHW 30%	253.1 (2.8)	287.12 (0.8)	-	8.3 (0.7)
Chitin 30%	264.1 (2.9)	289.6 (0.3)	397.2 (2.2)	4.1 (0.0)

**Table 3:** Characterisation by tensile test. Standard deviation is given in parentheses.

Sample	Tensile Strength at Break (MPa)	Elongation at Break (%)	Modulus (GPa)
Neat PHBV	34.5 (2.6)	13.9 (6.3)	1.7 (0.1)
SD 13%	39.1 (0.8)	3.8 (0.4)	1.9 (0.2)
SD 30%	46.2 (2.4)	2.9 (0.3)	2.7 (0.9)
Chitin 13%	37.2 (1.8)	4.5 (0.3)	1.9 (0.2)
Chitin 30%	32.0 (5.4)	2.0 (0.3)	1.7 (0.1)
HHW 13%	31.9 (4.0)	4.7 (1.2)	1.7 (0.2)
HHW 30%	28.3 (4.9)	3.8 (1.1)	1.7 (0.1)

**Table 4:** Amounts of ordered and disordered PHBV as obtained from the PHBV methyl area of the CPMAS NMR spectra.

Before	% of Total PHBV		After	% of Total PHBV	
Degradation	Ordered	Disordered	Degradation	Ordered	Disordered
neat PHBV	62.6	37.4	neat PHBV	61.1	38.9
13% SD	57.4	42.6	13% SD	68.4	31.7
13% chitin	58.9	41.1	13% chitin	62.0	38.0
13% HHW	62.8	37.2	13% HHW	79.3	20.7

## Data Availability

Data are contained within the article and Supplementary Materials.

## Supplementary Materials

The following supporting information can be downloaded at: https://www.mdpi.com/article/10.3390/ma17030768/s1, Figure S1: Characterisation by DSC (second heating) of neat PHBV (top), 30% chitin, 30% SD and 30% HHW (bottom); Figure S2: Thermogravimetric analysis of the neat polymer as well as with 30% loading of the organic waste; Figure S3: Tensile strength at break for the neat polymer as well as with 30% loading of the organic waste.
