# Supplementary material for: Development and Characterisation of Composites Prepared from PHBV Compounded with Organic Waste Reinforcements, and Their Soil Biodegradation
Source: Materials (Basel). 2024 Feb 5;17(3):768. doi: 10.3390/ma17030768 (PMC10856691; doi:10.3390/ma17030768)
Supplement: Supplementary file 1 [file materials-17-00768-s001.zip › materials-2804952-supplementary.pdf]

Supplementary File

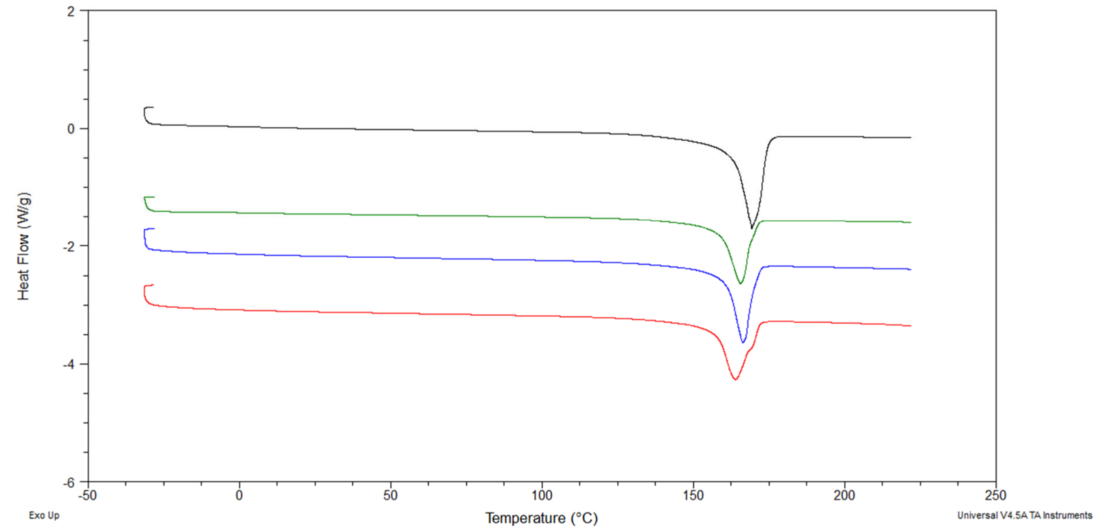

Figure S1. Characterisation by DSC (second heating) of neat PHBV (top), 30% chitin, 30% SD and 30% HHW (bottom).

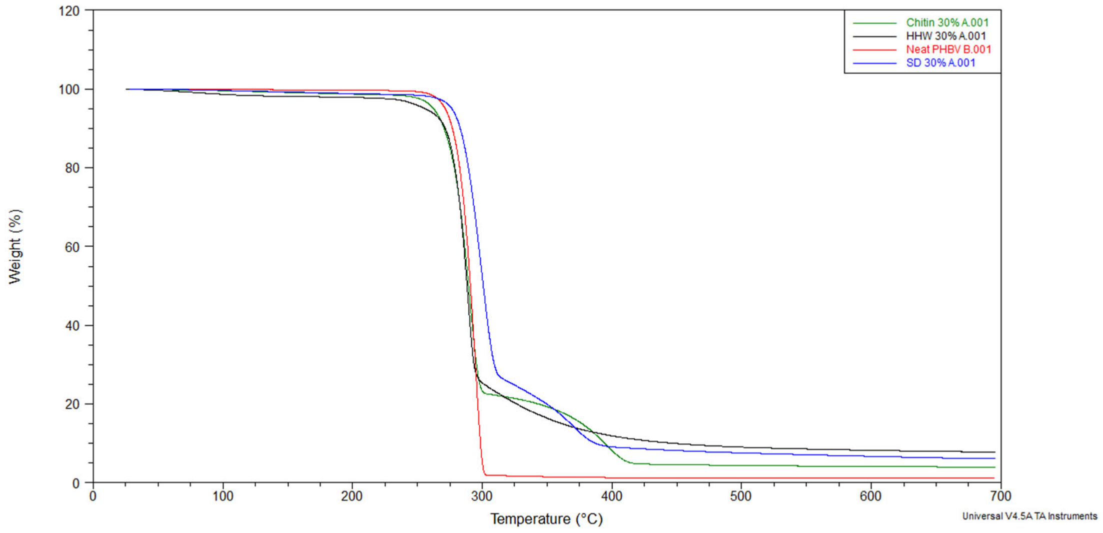

Figure S2. Thermogravimetric analysis of the neat polymer as well as with 30% loading of the organic waste.

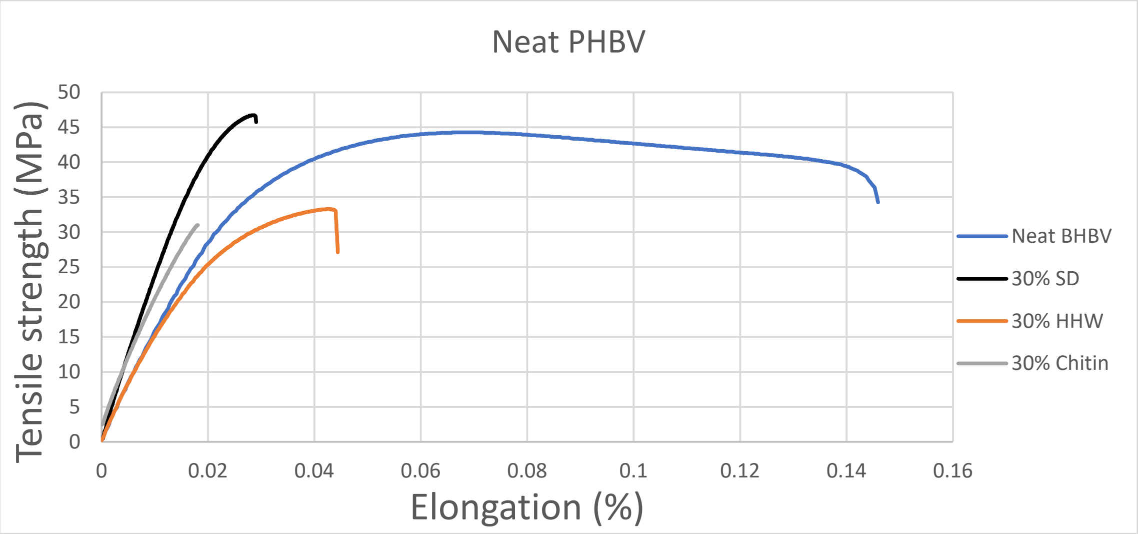

Figure S3. Tensile strength at break for the neat polymer as well as with 30% loading of the organic waste.
